# Baculovirus Genetic Diversity and Population Structure

**DOI:** 10.3390/v17020142

**Published:** 2025-01-22

**Authors:** Miguel López-Ferber, Primitivo Caballero, Trevor Williams

**Affiliations:** 1 HSM, University Montpellier, IMT Mines Ales, CNRS, IRD, 30319 Alès, France; 2 Institute for Multidisciplinary Research in Applied Biology, Universidad Pública de Navarra, 31006 Pamplona, Spain; pcm92@unavarra.es; 3 Departamento de Investigación y Desarrollo, Bioinsectis SL, 31110 Noain, Spain; 4 Instituto de Ecología AC (INECOL), Xalapa, Veracruz 91073, Mexico

**Keywords:** nucleopolyhedrovirus, granulovirus, Lepidoptera, genotype interactions, bioinsecticide, pest resistance

## Abstract

Baculoviruses can naturally regulate lepidopteran populations and are used as biological insecticides. The genetic diversity of these viruses affects their survival and efficacy in pest control. For nucleopolyhedroviruses, occlusion-derived virions and the occlusion body facilitate the transmission of groups of genomes, whereas this is not the case for granuloviruses. We review the evidence for baculovirus genetic diversity in the environment, in the host insect, and in occlusion bodies and virions. Coinfection allows defective genotypes to persist through complementation and results in the pseudotyping of virus progeny that can influence their transmissibility and insecticidal properties. Genetic diversity has marked implications for the development of pest resistance to virus insecticides. We conclude that future research is warranted on the physical segregation of genomes during virus replication and on the independent action of virions during infection. We also identify opportunities for studies on the transmission of genetic diversity and host resistance to viruses.

## 1. Introduction

Baculovirus structure and infection cycle

The *Baculoviridae* is a large family of insect-specific viruses distributed across four genera [1]. Within this family, nucleopolyhedroviruses (genus *Alphabaculovirus*) and granuloviruses (genus *Betabaculovirus*) lethally infect Lepidoptera and have an established history as the active ingredient for biological insecticides [2], and as biotechnological factories for protein production [3]. The genetic diversity of these viruses affects their survival, their evolvability, and their efficacy in pest control.

**Figure 1:**
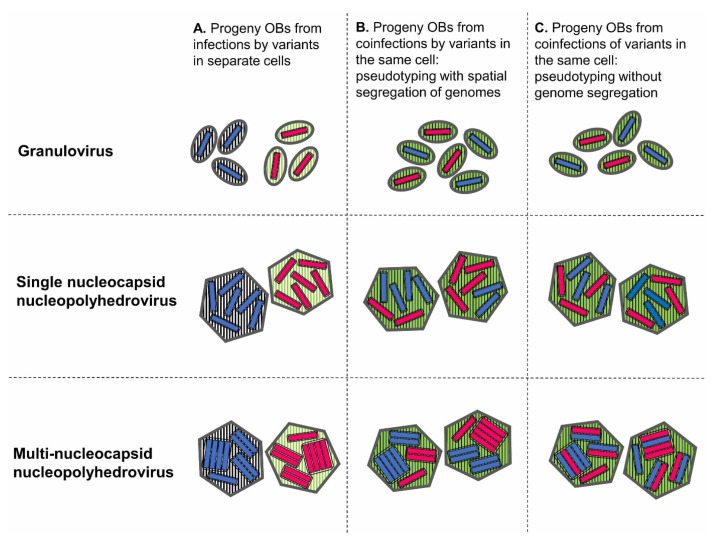
Occlusion body (OB) structure in relation to genetic diversity and the results of coinfection. Genotypic variants are represented as blue or pink genomes, encoding black hatched or green hatched proteins, respectively. If infections occur in separate cells, the content of each OB is homogeneous (column A). For granuloviruses, each genome is enveloped and occluded individually in an OB. Genotypic variants of granuloviruses that replicate in coinfected cells produce progeny with a mixed-variant pseudotype (mixed black and green hatching) but are occluded separately (column B, C). For single-nucleocapsid nucleopolyhedroviruses, single genomes are occluded in groups within each OB. Coinfection results in the pseudotyping of the progeny, with or without spatial segregation of genomes during replication and assembly (columns B, C). For multi-nucleocapsid nucleopolyhedroviruses, genomes are enveloped in groups comprising between one and many occlusion-derived virions (ODVs). Coinfection would result in the pseudotyping and segregation of virus progeny among ODVs if replication and assembly processes were spatially separated in the cell nucleus (column B), but this is uncertain. In the absence of segregation, progeny would produce genotypically diverse ODVs and OBs (column C).

Baculoviruses comprise a circular genome of double-stranded DNA (80–180 kbp) within a rod-shaped nucleocapsid. The nucleocapsids are enveloped singly or in groups by a lipid and protein membrane to form occlusion-derived virions (ODVs) (Figure 1). Nucleopolyhedrovirus ODVs are occluded in groups within a crystalline matrix of polyhedrin to form polyhedral occlusion bodies (OBs) in the cell nucleus, whereas granulovirus ODVs are occluded singly in a matrix of granulin to form ovoid granule-shaped OBs in the cytoplasm and degraded nucleus of the cell [4].

**Figure 2:**
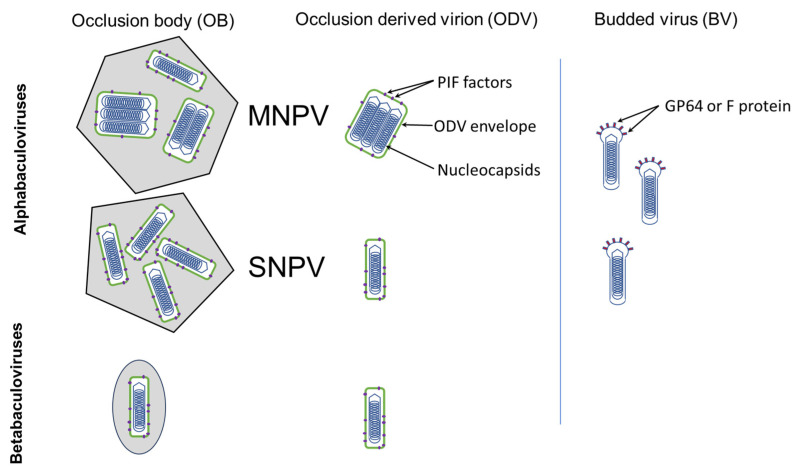
Structure of alphabaculoviruses (lepidopteran nucleopolyhedroviruses) and betabaculoviruses (lepidopteran granuloviruses). Following ingestion, occlusion bodies (OBs) dissolve and liberate occlusion-derived virions (ODVs). The ODVs infect midgut cells due to the presence of per os infectivity factors (PIF factors; purple ellipses) that link and fuse the virion membrane to the brush border of midgut cells. ODVs carry a single nucleocapsid in granuloviruses and single-enveloped nucleopolyhedroviruses (SNPVs). In contrast, each ODV can carry between one and several nucleocapsids in the multi-nucleocapsid nucleopolyhedroviruses (MNPVs). A different morphotype, the budded virus (BV), is produced in infected insects by the budding of individual nucleocapsids through the cellular cytoplasmic membrane. This BV carries fusion proteins of GP64 or envelope fusion protein (F protein), which allow infection of other cells in the larva (red spikes).

Horizontal transmission mainly occurs when larvae consume foliage contaminated with OBs. During primary infection, the OBs dissolve, releasing ODVs that cross the peritrophic membrane and infect midgut epithelial cells [5]. Secondary infections are mediated by budded viruses (BVs) that disperse in the hemocoel to infect the cells of other organs and tissues (Figure 2). Following replication, progeny nucleocapsids leave the cell as BVs early in infection but are retained later to form ODVs that are occluded into progeny OBs. Baculovirus replication has been reviewed in detail [6,7]. Nucleopolyhedrovirus-killed late-instar larvae can produce approximately 10^9^ OBs (each containing dozens of virus genomes), whereas granulovirus-killed larvae produce 10- to 100-fold more OBs, although each OB contains a single-virus genome.

Insect populations are susceptible to epizootics of baculovirus disease when they reach high densities that favor efficient transmission of these viruses, particularly in ecosystems in which larvae feed on exposed plant structures close to the soil surface [8]. This is due to the facility with which OBs can move between the soil reservoir and the plant surfaces on which transmission can occur. OBs can persist in a viable state for months or years in the soil environment, which contrasts with a persistence of hours or days on plant foliage exposed to solar radiation [9].

### 1.1. Variation in the Virus Genome

Nucleotide sequence variation mostly consists of indels (insertions and deletions), single-nucleotide polymorphisms (SNPs), and recombination events, all of which provide mechanisms for evolvability when viruses are faced with changes in their host or wider environment. This variation is not randomly distributed in the genome and affects regulatory regions and specific genes [10,11,12,13,14,15,16,17,18,19].

The DNA polymerases of baculoviruses have proofreading activity [20], resulting in ~1 × 10^−7^ substitutions per nucleotide in Autographa californica multiple nucleopolyhedrovirus (AcMNPV) [21]. However, due to the high number of genome copies, SNPs are frequently detected in baculovirus populations [22,23,24], ranging between 51 and 475 in Cydia pomonella granulovirus (CpGV) and 94 SNPs on average in the genome of AcMNPV [22,25]. Duplication events have also been described, both in coding regions [26] and in the *hrs* that act as transcription regulators and origins of replication. In addition, recombination has been observed during replication with coinfecting genomes of the same or different virus species [12,27,28,29] or with host genomic elements and transposons [30,31,32].

### 1.2. Variation in the Virus Population

Variation in genomic sequences is reflected in the appearance of “genotypic variants” in natural baculovirus isolates, but the observed variation largely depends on the methodology used. Cloning techniques, such as cell culture or in vivo cloning, do not allow all the variants to be amplified [33]. Replication in cell culture can also result in the production of variants with deletions in non-essential genes [34,35]. As such, studies on cloned variants may not accurately reflect the genotypic composition of a particular natural isolate [36].

Some of the variants present in field-collected isolates of nucleopolyhedroviruses may present large deletions in the genome [37,38,39], or disruption to genes involved in virulence [40], whereas others may be unstable during replication and represent a source of additional genetic diversity [41,42].

Restriction endonuclease analyses often demonstrate that isolates from diseased insects collected at the same location, or at different sites and times, are polymorphic for restriction fragment length and frequently present submolar bands that reflect their genotypic heterogeneity [43,44]. Such observations are more common in nucleopolyhedroviruses than in granuloviruses, although with the increasing use of next-generation sequencing, the presence of genotypic heterogeneity is also being recognized in granulovirus isolates [45].

### 1.3. Variant Interactions Affect Phenotype

Natural virus isolates almost invariably differ in insecticidal traits such as OB pathogenicity (measured as lethal dose metrics), speed of kill, and OB production when tested against a particular insect colony [39,46], across different host colonies [47], or in colonies of different host species [48]. Individual variants also show clear differences in their phenotype that influence their horizontal and vertical transmission [49], or their ability to replicate in a given host genotype [50,51].

Genotypic variants interact within the baculovirus population, and these interactions can increase or decrease the fitness of the population. For example, nine genotypic variants present in a Nicaraguan isolate (SfNIC) of the Spodoptera frugiperda nucleopolyhedrovirus (SfMNPV) were cloned, and each one was compared to the natural isolate. Significant differences were observed among variants in terms of OB pathogenicity and speed of kill. However, the frequency of each variant in the virus population was stable over time and generated a population phenotype that combined high OB pathogenicity but attenuated speed of kill, which resulted in high OB production [52].

A different interaction was detected in a commercial isolate of Spodoptera exigua MNPV (SeMNPV-SeUS2) [38]. However, in this case, the presence of one variant reduced the pathogenicity of mixed-genotype OBs, indicating that it was a cheater genotype that contributed negatively to virus transmission [33].

## 2. Mechanisms and Processes Affecting Diversity

Both the physical structure and the infection cycle of baculoviruses influence the transmission of genotypic diversity. Nucleopolyhedroviruses and granuloviruses show marked differences in some stages of the infection cycle, which will be highlighted when examining each level of diversity.

### 2.1. In the Environment

The OB structure allows baculoviruses to persist for months or years in the environment outside their host. Virus diversity present in the environment is inferred by collecting field-infected larvae or by feeding plants or soil samples to laboratory-reared insects [53], but next-generation sequencing of environmental DNA has not yet been applied to the characterization of genotypic diversity in baculoviruses from environmental samples. Importantly, the presence of viral DNA in environmental samples does not provide information on the viability of those genomes, which can be determined only by bioassay.

### 2.2. In the Host Organism

Lepidopteran larvae that ingest nucleopolyhedrovirus OBs are often infected by multiple genotypes [36]. This may be due to the consumption of several OBs on contaminated foliage, by consumption of a single OB containing a diversity of genotypes [54], or by de novo generation of diversity from a single unstable variant [41,42]. In some cases, only one genotype is detected in each larva, even in epizootic conditions in which each insect is likely to have consumed more than one OB, suggesting that selection for certain genotypes occurs very early in the infection process [55]. In granuloviruses, most OBs contain a single genome. Accordingly, multiple OBs have to be consumed to result in a genetically diverse infection, which is more likely at high OB densities or when the duration of the feeding period is extended [56]. The presence of more than one variant in a single larva has been observed in CpGV natural isolates from individual larvae [57] and in insects from virus-treated orchards [56].

In situations in which larvae consume multiple OBs, the probability of coinfection by different viruses increases. Natural coinfection of lepidopteran larvae by nucleopolyhedroviruses and granuloviruses is occasionally observed in field-collected larvae of various species of hosts [58], whereas natural coinfection by different nucleopolyhedroviruses is less common and limited to particular pathosystems, such as that of the multi-nucleocapsid and single-nucleocapsid viruses in *Thysanoplusia orichalcea* [59] and *Trichoplusia ni* [60] or the CfMNPV and the defective helper virus CfDEFNPV that infect *Choristoneura fumiferana* [61]. In other cases, distinct viruses have been isolated from the same host in a particular region, but natural coinfection has not been observed. One example is that of the nucleopolyhedroviruses that infect *Rachiplusia nu* in Argentina [62,63], possibly because coinfection is difficult to detect and is often overlooked in the absence of molecular studies or because one virus is capable of excluding or suppressing a second infection in the early stages of coinfection [64].

The midgut is an important filter for defective variants and acts as a genetic bottleneck of particular importance when insects consume low doses of OBs [65,66]. The number of OBs responsible for establishing an infection has been estimated at between 1.3 and 6.3 [67]. Accordingly, even low doses of OBs can result in multiple foci of infection, each comprising potentially different genotypes [68].

It is possible that covert infections of the host are also genotypically diverse, and this diversity might be transmitted vertically to the host’s offspring [4]. There is, however, some evidence that vertically transmitted variants are genetically distinct from variants that are transmitted horizontally [49,69,70]. The genetic diversity of latent and sublethal infections has not been the subject of scrutiny in baculoviruses, but given the probable bottleneck that this route imposes, opportunities for the transmission of genotypic diversity seem likely to vary between viruses that transmit transovarially (within the egg) and those that adopt transovum transmission, by surface contamination of the egg [71].

### 2.3. In the Cell

A second bottleneck occurs during the secondary infection mediated by BVs in cells of the organs and tissues within the hemocoel of the host. Coinfection of cells by different BVs is tightly controlled [72]. Synchronous coinfection with two genotypes of AcMNPV reached 95% of cells, but this was reduced to less than 20% when a delay of 16 h was set between the initial infection and subsequent infection by other virions arriving at the cell surface, a process known as superinfection exclusion [73]. As a result, early in host colonization, when BVs are scarce, cells are likely to be infected by just one or two virions, whereas later in infection, when BVs are abundant, cells are infected by an average of four or five virions in the *Trichoplusia ni*–AcMNPV pathosystem [74]. In consequence, the dynamics of BV production is expected to have a marked impact on the diversity of variants present in progeny OBs. Recent studies also suggest that the efficacy of superinfection exclusion is sensitive to the cell cycle, at least in latently infected *S. exigua* cells (Se301) [75].

Importantly, genomes that replicate together in a cell compete for host resources, but also share a common pool of transcription products that can be considered as public goods. The use of proteins from the shared pool has notable consequences. First, all the progeny viruses will have a shared pseudotype, irrespective of the genotype carried within their nucleocapsids (Figure 1, column B, C). This means they are likely to exhibit a similar infectious phenotype because they share the *cis*-acting proteins that are physically associated with virus particles and that have various roles in the early events of infection. Second, the production of essential factors by a complete genotype allows defective variants to acquire these factors for their own progeny, i.e., complementation. The transmission of defective genotypes is therefore dependent on frequent coinfection with complete genotypes.

### 2.4. In the Occlusion Body

The OB structure of nucleopolyhedroviruses is a clear example of a collective infectious unit [76], in which tens of virions are occluded in each OB [77]. Although the aggregation of virus particles in groups reduces the overall number of infectious units, this social transmission strategy can be advantageous in alleviating the genetic bottleneck during midgut infection [78]. In contrast, in granuloviruses, only a small fraction of the OBs may contain more than one nucleocapsid [79,80].

The first studies on the co-occlusion of variants inferred a physical association of variants [81,82]. More convincing evidence was provided by analyzing insects that consumed a single OB. Between one third and one half of insects acquired a genotypically diverse infection, involving between two and five variants from a single OB [54,83]. Recently developed methods, such as laser capture microdissection [84] and sequence-dependent genome autofluorescence [85], are likely to prove valuable for characterizing the composition of individual OBs.

### 2.5. In the Occlusion-Derived Virion

Single-nucleocapsid nucleopolyhedroviruses have one nucleocapsid and one genome wrapped within each ODV (Figure 1). In contrast, multi-nucleocapsid nucleopolyhedroviruses have ODVs that comprise between one and tens of nucleocapsids that are co-enveloped together within each ODV. The number of nucleocapsids per ODV varies in different nucleopolyhedroviruses and also across different isolates of the same virus species [86]. In AcMNPV, for example, 90% of the ODVs are of the multi-nucleocapsid morphotype, comprising 2–10 nucleocapsids per ODV and an average of six nucleocapsids per ODV [66]. The number or morphotype of ODVs occluded within OBs has been demonstrated to have a genetic basis [52,87,88,89,90], although cellular factors and cell physiology are also influential [91,92]. Despite its importance in the transmission of genetic diversity, this is an aspect of the insecticidal phenotype that is rarely considered in isolate characterization studies.

The simultaneous delivery of various nucleocapsids into midgut cells by multi-nucleocapsid ODVs may have direct effects on virus fitness. First, a fraction of the nucleocapsids can immediately be repackaged and exported as BVs without the need for virus replication [7,93]. It appears that single- and multiple-type ODVs have a similar probability of infecting midgut cells, but for the same number of nucleocapsids, envelopment in single ODVs creates far more individual virions. However, multi-nucleocapsid ODVs appeared to be capable of establishing secondary infections faster than cells infected by single-nucleocapsid ODVs, which favors the rapid establishment of a systemic infection that is independent of the fate of the primary infected cell [93]. Second, as virus genomes carry an arsenal of genes capable of blocking apoptosis and global protein shutdown responses of the host cell, the simultaneous delivery of multiple copies of these genes may increase the virus’ ability to overcome the cell’s innate immune response during the earliest stages of infection [94,95,96]. Third, among the virus nucleocapsids that migrate to the nucleus, those with UV-damaged genomes would be able to recover viability through recombination with coinfecting genomes [91]. Fourth, SNPV and MNPV viruses can differ in their fitness depending on the host plant, as observed in the tussock moth *Orgyia pseudotsugata*, which feeds on various species of firs (*Abies* spp.) in North America. The single and multiple morphotypes coexist but differ in infectiousness, speed of kill, and in their ability to avoid detection by host larvae feeding on different species of OB-contaminated foliage [92].

Evidently, the diversity of genotypic variants present within ODVs depends on the number and diversity of the BVs that infected the host cell in which they were assembled. When cultured cells were inoculated with highly diluted suspensions of SfMNPV ODVs, mixtures of genotypes were detected at higher-than-expected frequencies, indicating that several genotypes were present in individual ODVs [54]. More recently, the presence of the genomes of different virus species were demonstrated to be co-enveloped within individual ODVs that were produced as a result of mixed-virus infection of a shared host [83,97].

A study involving the use of recombinant viruses concluded that midgut coinfection was approximately ten-fold less common than expected assuming a random assortment of genomes in OBs [98]. Several factors may have contributed to this finding, in which a midgut reporter gene assay was employed to determine the presence of coinfected cells. The role of inoculum nucleocapsid repackaging and export may be particularly relevant given that the authors observed midgut infection foci at 72 h post-inoculation, which is notably later than the cell sloughing response to infection [94]. Alternatively, few cells may have suitably expressed reporter genes under the control of very late promoters. These authors argue that during replication in coinfected cells, nucleocapsids containing the genomes of different variants are spatially segregated into different ODVs, so that midgut infections originating from mixed-variant ODVs are uncommon [98]. These findings require confirmation and emphasize the need to better understand the physical arrangement of genotypic variants at the OB and ODV levels. In this respect, the recently developed single-cell and single-nucleus RNA sequencing technologies could provide valuable insights into the genetic composition of ODVs involved in the primary infection process [99].

It has been suggested that due to the high number of genomes produced in infected cells, a compartmentation mechanism could exist to limit contact between them [100]. This proposed mechanism would link genome replication and nucleocapsid formation, and perhaps membrane envelopment and OB condensation [7]. Under such conditions, the condensation of genetically diverse OBs would be limited.

## 3. Processes That Favor Genotypic Diversity

Genetic diversity in virus populations is selectively advantageous and has ecological and evolutionary benefits to these viruses. Diversity is maintained through four main processes.

### 3.1. Trade-Offs Between Components of Virus Fitness

Trade-offs between components of virus fitness mean that variants that differ in specific traits can be favored in different individuals (or different tissues) or when transmission opportunities vary. A well-recognized trade-off involves the negative correlation between the speed of kill and the number of progeny OBs that are produced in each infected host. A faster speed of kill could allow more cycles of horizontal transmission in a given period but results in the production of fewer infectious OBs in each infected host, which reduces the probability of transmission [101,102]. The environmental persistence of OBs was found to be negatively correlated with the transmission rate of 16 isolates of LdMNPV [103]. A trade-off was also observed between the transmission rate and variation in transmission, so that a strain with a lower and less variable transmission rate could coexist in a host population with a strain having a higher but more variable transmission rate [102]. Indeed, trade-offs in virus phenotypes that affect transmissibility mean that phenotypically diverse virus strains are likely to provide better control of host populations than individual genotypes alone [104].

### 3.2. Interactions Between Virus Genotypes

The interactions between and among genotypes can involve *cis*- or *trans*-acting factors. The *cis*-acting factors require coinfection of cells, whereas the *trans*-acting factors do not. For example, the variants that produce enhancin factors that degrade the peritrophic matrix do so to the benefit of all variants present in the inoculum, which is an example of a *trans*-acting interaction that does not depend on a physical association of the variants. Such *trans*-acting interactions can even occur between different species of viruses [105].

Evidence for interactions between and among genotypes comes from two types of inocula, namely OBs from different sources that are mixed and used as inoculum, or mixtures of variants that have replicated in the same cell and become co-occluded in mixed-genotype OBs. When single-genotype OBs are mixed and used to inoculate larvae, the host mortality response, speed of kill, and progeny OB yields can be affected, although the mechanisms involved in these interactions are mostly unclear [37,106].

When OBs are produced in a coinfected cell, all progeny ODVs share the pool of viral proteins. In certain cases, virion pseudotyping can alter the infectivity phenotype and extend the host range of these viruses [83,97]. The factors that provide entry into host cells are *cis*-acting factors, physically associated with the virus particle, such as the per os infectivity factors (PIFs) that compose the fusion mechanism between the ODV envelope and the midgut cell membrane [107].

When mixtures of variants are co-occluded to produce mixed-genotype OBs, significant changes in the host mortality response, speed of kill, and progeny OB yield have been observed in several pathosystems [37,52,108,109,110,111]. Importantly, the direction and magnitude of these changes cannot be readily predicted from the phenotypic traits of the component variants, although in some cases, such as the *cis*-acting PIF-1 factor in Sf-NIC variants, manipulation of gene expression can provide useful insights into the mechanisms underlying variant interactions [112].

In theory, *cis*-acting factors might also act during systemic infection of the host, assuming that host cells are infected by several BVs. For example, CpGV-M does not kill the larvae of type I resistant codling moth, whereas CpGV-R5 is fully infectious and lethal. However, CpGV-M can infect and replicate in resistant insects previously infected by CpGV-R5 [113]. The “helper” effect of CpGV-R5 cannot be substituted by other baculoviruses that are able to replicate in codling moth. This finding brought into question the hypothesis of independent infection of variants and suggested the possible existence of a viral communication system, similar to the *trans*-acting arbitrium viral peptide produced following phage infection in bacteria [114]. Moreover, mixtures of OBs of both variants have higher pathogenicity to both susceptible and resistant insect colonies, when compared to each variant alone [115,116]. The mechanisms involved in this apparent cooperation remain uncertain.

### 3.3. Differential Selection for Genotypes

Genotypic variants often vary in their capacity to infect and replicate in hosts. As a result, transmission experiments in homologous or heterologous hosts frequently result in changes in the prevalence of particular variants [117,118,119,120]. Indeed, quantitative PCR techniques are currently being developed to examine the transmission and persistence of genetic diversity in the SfMNPV Nicaraguan isolate [121].

Genetic drift was quantified in *S. exigua* larvae inoculated with an equal ratio of two AcMNPV genotypes and resulted in a roughly 100-fold difference in the ratio of genotypes that replicated in infected individuals, likely due to a combination of stochastic variation in the primary infection and variation in host susceptibility to infection [66]. Similarly, genetic drift caused by transmission bottlenecks and variation in virus replication within hosts was identified in a field-collected sample of 143 LdMNPV-infected *Lymantria dispar* larvae [122]. High levels of heterogeneity in host susceptibility to infection means that a fraction of the host population is highly prone to infection, which favors the transmission of inoculum with high genetic diversity, even at low inoculum concentrations [123].

Testing families of *L. dispar* against a range of LdMNPV isolates provided evidence for host genotype × virus genotype interactions, which promoted variation in both host and virus populations [124]. As no host family is resistant to all genotypes, and no isolate is highly infectious to all host families, genotype × genotype interactions may also promote negative frequency dependent selection, which could explain how rare virus genotypes are able to persist and seem to be a feature of baculovirus populations [120]. Such interactions are particularly apparent in the populations of *C. pomonella* that show different types of resistance to CpGV [125] and vary in their susceptibility to different CpGV genotypes [116].

### 3.4. Genotype × Environment Interactions

Variants perform differently in terms of transmission or persistence in distinct environments. For example, certain variants of SeMNPV were particularly prevalent in greenhouse soil substrate, even soils with an alkaline pH, suggesting that a fraction of the virus population may be better adapted to persist in the soil reservoir during periods when the host is absent [126]. Food plants can strongly influence virus transmission as plants affect the insect’s immune response, virus interactions with plant chemicals, and the composition of the gut microbiota [127,128]. As a result, the composition of the virus population may reflect the host’s feeding habits and the vegetation present in each locality [106,129,130].

## 4. Genetic Diversity in Biological Insecticides

As the genetic composition of insect populations fluctuates over time and space, their susceptibility to virus populations and individual variants varies [131], which poses a challenge in the choice of the most efficient isolate or variant for the production of biological insecticides. Mass production requires the continuous monitoring of the product quality, which is easier if the isolate is genetically homogeneous. Indeed, Lee and Miller [132] suggested that the use of cloned viruses or homogeneous isolates would facilitate quality control, but later, Lynn et al. [133] proposed the production of multiple independent clones that could be mixed in the final product to improve the efficacy of these products against genetically heterogenous pest populations. Currently, the selection of the active material of virus-based insecticides usually involves characterization of the insecticidal phenotype of a range of natural isolates. The most suitable isolate is then subjected to formulation and field efficacy tests and used to produce the desired insecticidal product [2]. In some cases, these products can comprise mixtures of different virus species, likely produced in different host species, to create insecticides with increased host range [134].

The use of recombinant DNA technology to improve the efficacy of virus-based insecticides has attracted considerable attention over the past few decades [135]. This requires the initial selection and cloning of a genotypic variant. However, the rapid demise of recombinant-infected larvae makes OB production challenging for these viruses [136], so that all commercial insecticides are currently based on unmodified viruses.

The spread of type I resistance to CpGV in codling moth populations highlighted the need for genetic heterogeneity in virus-based insecticides. To control resistant codling moth, several of the currently available products appear to comprise mixtures of at least two genotypic variants, which increases their efficacy [115,116]. Accordingly, virus insecticides composed of various genotypic variants are likely to prove more sustainable as pest control products, but maintaining stable frequencies of each genotype is a challenge in their production. The methods required to produce uniform batches of genotypically complex OBs will vary according to the virus, the uniformity of the insect colony, and the type of interactions that can occur among variants.

A recent approach to improving virus-based insecticides has focused on producing laboratory-designed mixtures of genotypic variants with desirable insecticidal properties. This involves the coinfection of larvae with mixtures of variants in varying proportions to produce mixed-genotype OBs with increased pathogenicity compared to natural isolates or the component genotypic variants [137]. This approach has been applied to the development of SeMNPV [138] and HearNPV [109], whereas co-occlusion of mixtures of variants did not improve the pathogenicity of AgMNPV or SfMNPV OBs over that of natural isolates [37,109]. Serial passage of co-occluded preparations in larvae can affect the prevalence of genotypes in the mixture, resulting in an additional increase in OB pathogenicity [109]. Although increased pathogenicity has been the primary objective of these studies, the same approach could be applied to optimizing speed of kill or OB production characteristics [27].

The concept of co-occlusion was taken a step further with the observation that in some cases it was possible to co-occlude mixtures of different nucleopolyhedroviruses. For this, a shared host is required in which both viruses can replicate. A fraction of the viruses that coinfect and replicate in the same cell are enveloped together in mixed-virus ODVs [97]. For example, mixtures have been produced for AcMNPV mixed with SfMNPV or MbMNPV, SfMNPV in mixtures with MbMNPV or SeMNPV, and mixtures of the single-nucleocapsid HearNPV and the multi-nucleocapsid HearMNPV, which are not closely related and show divergent host range characteristics [83,97]. The mixed-virus OBs produce lethal infections in both the original hosts, but the replication of each virus in the heterologous host is marginal in the systems studied. This suggests that following application of the co-occluded preparations in the field, a heterologous virus would likely be eliminated from mixed-virus OBs within a few cycles of insect-to-insect transmission, thus ensuring the biosafety of co-occluded insecticides. In a separate laboratory study, SeMNPV OBs were found to harbor co-occluded iflaviruses. The ingestion of these OBs resulted in the transmission of both types of viruses and was associated with significant variation in their insecticidal properties, although the degree to which this phenomenon occurs in nature remains unclear [139]. Nonetheless, when applied to mixtures of nucleopolyhedroviruses, the co-occlusion technology could pave the way to new virus insecticides with a host range based on the needs of growers of crops attacked by combinations of lepidopterous pests [137].

### Risks of Resistance

Heterogeneity in insect susceptibility to virus infection means that repeated application of high doses of viral OBs over large areas selects for resistance in pest populations. This has been demonstrated in laboratory colonies of insects exposed to repeated challenges of granuloviruses [140], or nucleopolyhedroviruses [20,141,142,143,144], with resistance ratios of approximately ten-fold to many thousand-fold, depending on the host species and the number of generations exposed. Resistance can also appear in natural insect populations during epizootics of disease, but to a lesser extent [36]. No notable resistance issues arose during two decades of widespread use of genotypically diverse AgMNPV-based products in soybean crops in Brazil [145], whereas resistance was observed in laboratory colonies challenged with a single isolate of this virus [141].

Insecticides based on CpGV for control of the codling moth in Europe were all developed using the CpGV-M isolate originally collected in Mexico [146], which presents undetectable variability and may be considered as clonal. Beginning in 1988, the use of CpGV-M-based products in European countries progressively increased to >100,000 Ha/year, but failures in pest control from 2003 were due to the development of resistance [147,148]. Today, CpGV genotypic variants are classified in seven phylogenetic groups [25,149], and a total of five types of resistance to CpGV have been identified in *C. pomonella* populations [125]. CpGV variants are able to bypass one or more types of resistance mechanisms but are fully or partially blocked by others [25,150].

Possible approaches for resistance management in codling moth involve either the sequential use of products containing a single variant, or the use of products comprising a mixture of variants. Selection of the appropriate control strategy needs to consider not only the independent action of each isolate, and the fitness cost of resistance to each isolate for the host, but also the result of the interaction between isolates. The observed increase in efficacy of genotypically diverse CpGV suggests that the second approach might be more sustainable [115,116]. However, as coinfection by granuloviruses implies ingestion of more than one OB, this will require increasing the dose of OBs applied in the field, with an associated increase in production costs.

## 5. Future Issues

The growing recognition of the importance of genetic diversity in baculovirus populations has highlighted four principal issues that merit the attention of researchers in virology and bioinsecticide development.

### 5.1. Physical Segregation of Variants

The spatial and physical processes within the nucleus that determine the production of nucleocapsids, their envelopment into ODVs, and OB condensation are not well established. Clearly, these processes are likely to influence the composition of ODVs and OBs that transmit genotypic diversity from insect to insect. The cell environment [19] and mutations in the viral DNA polymerase [151] have been implicated in affecting the proportions of single- and multi-nucleocapsid ODVs. This suggests that the rate of genome replication may have downstream effects on the enveloping of nucleocapsids singly or in groups. The assembly of nucleocapsids in association with microvesicle-rich regions of the nuclear virogenic stroma, from which ODV membranes may be derived, also appears likely to influence ODV composition but is poorly understood [92]. Spatial segregation of genomes during replication and encapsidation was recently implicated in low levels of midgut coinfection by AcMNPV recombinant viruses [97]. As this finding would appear to contradict the findings of others [137], the possibility that variant genomes are segregated among different ODVs merits closer examination.

### 5.2. Transmission of Diversity

It is clear that baculoviruses face genetic bottlenecks during horizontal transmission, initially to establish a primary infection in midgut cells, and during systemic infection of host cells that is restricted to a brief (~16 h) temporal window. Transovum or transovarial vertical transmission to offspring is likely to represent an additional bottleneck. We have a poor understanding of the magnitude of these bottlenecks, although attempts have been made to quantify the number of founder genomes following oral inoculation in some host-nucleopolyhedrovirus systems [66,99]. If the number of variants present in progeny OBs exceeds that of the founders, the additional diversity must have been generated during replication and there is indeed growing evidence for the de novo generation of variants [22,41,152].

The advantages of each occlusion strategy for nucleopolyhedroviruses (multiple virions in each OB) and granuloviruses (a single virion in each OB) are unclear, as the choice of strategy dictates the likelihood of coinfection (and the transmission of genetic diversity) at the organismal level. In general, granuloviruses have a narrower host range and are more specialized in infecting a particular host species than nucleopolyhedroviruses, so that the granulovirus strategy appears to be based on maximizing the likelihood of establishing infection from a single OB. In consequence, granuloviruses prove more successful than nucleopolyhedroviruses when the amount of leaf surface consumed by a larva is small, as is the case for insects that develop inside plant structures, in which opportunities for horizontal transmission are restricted in time and space. Notable examples include the tortricids *C. pomonella*, *Cryptophlebia leucotreta*, and *Epinotia* (*Crocidosema*) *aporema*, the gelechiids *Tecia solanivora* and *Phthorimaea operculella,* and the crambid *Diatraea saccharalis*. As such, the granulovirus strategy involves producing an exceptionally large number of highly infectious progeny OBs in each infected larva.

By contrast, the occlusion strategy involving groups of ODVs seen in nucleopolyhedroviruses is becoming elucidated through new sociovirology approaches that highlight the benefits of collective transmission in establishing infection, replication, and the ability to subjugate host defenses [78,153]. Nonetheless, a number of issues related to the social interactions of viruses have been highlighted including the role of defective (cheater) genotypes, the trade-offs that arise from optimizing within- and between-host transmissions, and the optimal group size for coinfecting genotypes, all of which are faced by nucleopolyhedroviruses [154].

An additional unresolved question focuses on the conditions under which the multi-nucleocapsid strategy is favored over the single-nucleocapsid strategy of genome delivery to midgut cells. As the number and distribution of nucleocapsids among ODVs is a virus-specific trait, this would suggest that this trait is optimized for the midgut characteristics and cell sloughing behavior of the host. It would be interesting to compare the success of single- and multi-nucleocapsid ODVs in viruses that are host-specific, such as SeMNPV, with those that encounter a broader range of potential hosts, such as AcMNPV and MbMNPV, in order to examine the correlation between inoculum repackaging and pass-through events and cell sloughing rate and whether this differs for viruses that differ in host specificity. ODV enrichment studies could prove useful for this type of study [94].

### 5.3. Host Resistance

As resistance is of key importance in pest control, it deserves close attention. Experience with CpGV proves that insects can develop resistance to single-genotype insecticides, but to date, no resistance has been observed to genotypically diverse insecticides under field conditions [155]. Indeed, virus diversity likely hinders the development of host resistance. In a laboratory study on *T. ni*, resistance was generally higher and developed faster when exposed to single variants than mixtures of variants or a natural isolate of AcMNPV [156]. In addition, various virus fitness components (productivity, virulence) appear to be independently selected, leading to different strategies in each host, which results in an increased virus population diversity [157].

Resistance often imposes a cost to host fitness, such as slower development or reduced body weight [155]. For example, fitness costs were more severe when the inoculum was genotypically diverse compared to the costs of exposure to single variants of AcMNPV [156]. Resistance costs were also higher on poor-quality diets [158,159,160], so we might predict higher host resistance and greater diversity in virus populations associated with high-quality food plants and the opposite on marginal food plants, despite the marked effects that food plants can have on the host immune response [161]. However, no cost was observed in codling moths for resistance to CpGV-M [162].

### 5.4. Independent Action of Virions

Finally, and importantly, the hypothesis underlying all previous studies is that of the independence of infection of each cell by ODVs and BVs. This assumption warrants scrutiny, as sequential infection of the codling moth by different genotypes of CpGV suggests the production of helper molecules that facilitate the action of other genotypes [113]. If confirmed, these findings could significantly change our understanding of the infection process and the functional importance of genotypic diversity in this family of viruses.

## References

[1] Harrison R.L., Herniou E.A., Jehle J.A., Theilmann D.A., Burand J.P., Becnel J.J., Krell P.J., van Oers M.M., Mowery J.D., Bauchan G.R. (2018). ICTV virus taxonomy profile: Baculoviridae. J. Gen. Virol..

[2] Moore S., Jukes M., Kogan M., Higley L. (2019). Advances in microbial control in IPM: Entomopathogenic viruses. Integrated Management of Insect Pests.

[3] van Oers M.M., Pijlman G.P., Vlak J.M. (2015). Thirty years of baculovirus–insect cell protein expression: From dark horse to mainstream technology. J. Gen. Virol..

[4] Williams T., Bergoin M., van Oers M.M. (2017). Diversity of large DNA viruses of invertebrates. J. Invertebr. Pathol..

[5] Erlandson M.A., Toprak U., Hegedus D.D. (2019). Role of the peritrophic matrix in insect-pathogen interactions. J. Insect Physiol..

[6] Blissard G.W., Theilmann D.A. (2018). Baculovirus entry and egress from insect cells. Annu. Rev. Virol..

[7] Rohrmann G.F. (2019). Baculovirus Molecular Biology.

[8] Fuxa J.R. (2004). Ecology of insect nucleopolyhedroviruses. Agric. Ecosyst. Environ..

[9] Williams T. (2023). Soil as an environmental reservoir for baculoviruses: Persistence, dispersal and role in pest control. Soil Syst..

[10] Craveiro S.R., Melo F.L., Ribeiro Z.M.A., Ribeiro B.M., Báo S.N., Inglis P.W., Castro M.E.B. (2013). Pseudoplusia includens single nucleopolyhedrovirus: Genetic diversity, phylogeny and hypervariability of the *pif-2* gene. J. Invertebr. Pathol..

[11] D’Amico V., Slavicek J., Podgwaite J.D., Webb R., Fuester R., Peiffer R.A. (2013). Deletion of *v-chiA* from a baculovirus reduces horizontal transmission in the field. Appl. Environ. Microbiol..

[12] de Brito A.F.D., Braconi C.T., Weidmann M., Dilcher M., Alves J.M.P., Gruber A., Zanotto P.M.D.A. (2016). The pangenome of the Anticarsia gemmatalis multiple nucleopolyhedrovirus (AgMNPV). Genome Biol. Evol..

[13] Harrison R.L. (2013). Concentration-and time-response characteristics of plaque isolates of Agrotis ipsilon multiple nucleopolyhedrovirus derived from a field isolate. J. Invertebr. Pathol..

[14] Kitchin D., Bouwer G. (2018). Significant differences in the intra-host genetic diversity of Helicoverpa armigera nucleopolyhedrovirus *dnapol* after serial in vivo passages in the same insect population. Arch. Virol..

[15] Martemyanov V.V., Kabilov M.R., Tupikin A.E., Baturin O.A., Belousova I.A., Podgwaite J.D., Ilynykh A.V., Vlassov V.V. (2015). The enhancin gene: One of the genetic determinants of population variation in baculoviral virulence. Dokl. Biochem. Biophys..

[16] Masson T., Fabre M.L., Pidre M.L., Niz J.M., Berretta M.F., Romanowski V., Ferrelli M.L. (2021). Genomic diversity in a population of Spodoptera frugiperda nucleopolyhedrovirus. Infect. Genet. Evol..

[17] Niz J.M., Salvador R., Ferrelli M.L., Sciocco de Cap A., Romanowski V., Berretta M.F. (2020). Genetic variants in Argentinean isolates of Spodoptera frugiperda multiple nucleopolyhedrovirus. Virus Genes.

[18] Thézé J., Cabodevilla O., Palma L., Williams T., Caballero P., Herniou E.A. (2014). Genomic diversity in european Spodoptera exigua multiple nucleopolyhedrovirus isolates. J. Gen. Virol..

[19] Xu Y.P., Cheng R.L., Xi Y., Zhang C.X. (2013). Genomic diversity of Bombyx mori nucleopolyhedrovirus strains. Genomics.

[20] Milks M.L., Myers J.H. (2000). The development of larval resistance to a nucleopolyhedrovirus is not accompanied by an increased virulence in the virus. Evol. Ecol..

[21] Boezen D., Ali G., Wang M., Wang X., van der Werf W., Vlak J.M., Zwart M.P. (2021). Empirical estimates of the mutation rate for n alphabaculovirus. PLoS Genet..

[22] Chateigner A., Bézier A., Labrousse C., Jiolle D., Barbe V., Herniou E.A. (2015). Ultra deep sequencing of a baculovirus population reveals widespread genomic variations. Viruses.

[23] Wennmann J.T., Radtke P., Eberle K.E., Gueli-Alletti G., Jehle J.A. (2017). Deciphering single nucleotide polymorphisms and evolutionary trends in isolates of the Cydia pomonella granulovirus. Viruses.

[24] McDougal V.V., Guarino L.A. (1999). Autographa californica nuclear polyhedrosis virus DNA polymerase: Measurements of processivity and strand displacement. J. Virol..

[25] Fan J., Wennmann J.T., Wang D., Jehle J.A. (2020). Novel diversity and virulence patterns found in new isolates of Cydia pomonella granulovirus from China. Appl. Environ. Microbiol..

[26] López-Ferber M., Argaud O., Croizier L., Croizier G. (2001). Diversity, distribution and mobility of bro gene sequences in Bombyx mori nucleopolyhedrovirus. Virus Genes.

[27] Barrera G.P., Belaich M.N., Patarroyo M.A., Villamizar L.F., Ghiringhelli P.D. (2015). Evidence of recent interspecies horizontal gene transfer regarding nucleopolyhedrovirus infection of *Spodoptera frugiperda*. BMC Genom..

[28] Croizier G., Ribeiro H.C.T. (1992). Recombination as a possible major cause of genetic heterogeneity in Anticarsia gemmatalis nuclear polyhedrosis virus wild populations. Virus Res..

[29] Simón O., Williams T., Possee R.D., López-Ferber M., Caballero P. (2010). Stability of a Spodoptera frugiperda nucleopolyhedrovirus deletion recombinant during serial passage in insects. Appl. Environ. Microbiol..

[30] Gilbert C., Chateigner A., Ernenwein L., Barbe V., Bézier A., Herniou E.A., Cordaux R. (2014). Population genomics supports baculoviruses as vectors of horizontal transfer of insect transposons. Nat. Commun..

[31] Loiseau V., Peccoud J., Bouzar C., Guillier S., Fan J., Gueli Alletti G., Meignin C., Herniou E.A., Federici B.A., Wennmann J.T. (2021). Monitoring insect transposable elements in large double-stranded DNA viruses reveals host-to-virus and virus-to-virus transposition. Mol. Biol. Evol..

[32] Yanase Y., Hashimoto Y., Kawarabata T. (2000). Identification of insertion and deletion genes in Autographa californica nucleopolyhedrovirus variants isolated from *Galleria mellonella*, *Spodoptera exigua*, *Spodoptera litura* and *Xestia c-nigrum*. Virus Genes.

[33] Serrano A., Williams T., Simón O., López-Ferber M., Caballero P., Muñoz D. (2013). Analogous population structures in two alphabaculoviruses highlight functional role for deletion mutants *Appl*. Environ. Microbiol..

[34] Erlandson M.A. (2009). Genetic variation in field populations of baculoviruses: Mechanisms for generating variation and its potential role in baculovirus epizootiology. Virol. Sin..

[35] Pijlman G.P., van den Born E., Martens D.E., Vlak J.M. (2001). Autographa californica baculoviruses with large genomic deletions are rapidly generated in infected insect cells. Virology.

[36] Cory J.S., Green B.M., Paul R.K., Hunter-Fujita F. (2005). Genotypic and phenotypic diversity of a baculovirus population within an individual insect host. J. Invertebr. Pathol..

[37] Barrera G., Williams T., Villamizar L., Caballero P., Simón O. (2013). Deletion genotypes reduce occlusion body potency but increase occlusion body production in a Colombian Spodoptera frugiperda nucleopolyhedrovirus population. PLoS ONE.

[38] Muñoz D., Castillejo J.I., Caballero P. (1998). Naturally occurring deletion mutants are parasitic genotypes in a wild-type nucleopolyhedrovirus population of *Spodoptera exigua*. Appl. Environ. Microbiol..

[39] Simón O., Williams T., López-Ferber M., Caballero P. (2004). Genetic structure of a Spodoptera frugiperda nucleopolyhedrovirus population: High prevalence of deletion genotypes. Appl. Environ. Microbiol..

[40] Ferreira B.C., Melo F.L., Silva A.M.R., Sanches M.M., Moscardi F., Ribeiro B.M., Souza M.L. (2019). Biological and molecular characterization of two Anticarsia gemmatalis multiple nucleopolyhedrovirus clones exhibiting contrasting virulence. J. Invertebr. Pathol..

[41] Aguirre E., Beperet I., Williams T., Caballero P. (2021). Generation of variability in Chrysodeixis includens nucleopolyhedrovirus (ChinNPV): The role of a single variant. Viruses.

[42] Baillie V.L., Bouwer G. (2012). High levels of genetic variation within Helicoverpa armigera nucleopolyhedrovirus populations in individual host insects. Arch. Virol..

[43] Redman E.M., Wilson K., Grzywacz D., Cory J.S. (2010). High levels of genetic diversity in Spodoptera exempta NPV from Tanzania. J. Invertebr. Pathol..

[44] Vickers J.M., Cory J.S., Entwisle P.F. (1991). DNA characterization of eight geographic isolates of granulosis virus from the potato tuber moth (*Phthorimaea operculella*) (Lepidoptera, Gelechiidae). J. Invertebr. Pathol..

[45] Fan J., Jehle J.A., Wennmann J.T. (2021). Population structure of Cydia pomonella granulovirus isolates revealed by quantitative analysis of genetic variation. Virus Evol..

[46] Aguirre E., Beperet I., Williams T., Caballero P. (2019). Genetic variability of Chrysodeixis includens nucleopolyhedrovirus (ChinNPV) and the insecticidal characteristics of selected genotypic variants. Viruses.

[47] Briese D.T., Mende H.A. (1981). Differences in susceptibility to a granulosis virus between field populations of the potato tuber moth, *Phthorimaea operculella* (Zeller) (Lepidoptera: Gelechiidae). Bull. Entomol. Res..

[48] Espinel-Correal C., López-Ferber M., Zeddam J.L., Villamizar L., Gomez J., Cotes A.M., Léry X. (2012). Experimental mixtures of Phthorimaea operculella granulovirus isolates provide high biological efficacy on both *Phthorimaea operculella* and *Tecia solanivora* (Lepidoptera: Gelechiidae). J. Invertebr. Pathol..

[49] Cabodevilla O., Ibañez I., Simón O., Murillo R., Caballero P., Williams T. (2011). Occlusion body pathogenicity, virulence and productivity traits vary with transmission strategy in a nucleopolyhedrovirus. Biol. Control.

[50] Berling M., Blachère-Lopez C., Soubabère O., Léry X., Bonhomme A., Sauphanor B., López-Ferber M. (2009). Cydia pomonella granulovirus (CpGV) genotypes overcome virus resistance in the codling moth and improve virus efficiency by selection against resistant hosts. App. Environ. Microbiol..

[51] Eberle K.E., Asser-Kaiser S., Sayed S.M., Nguyen H.T., Jehle J.A. (2008). Overcoming the resistance of codling moth against conventional Cydia pomonella granulovirus (CpGV-M) by a new isolate CpGV-I12. J. Invertebr. Pathol..

[52] Simon O., Williams T., López-Ferber M., Taulemesse J.M., Caballero P. (2008). Population genetic structure determines speed of kill and occlusion body production in Spodoptera frugiperda multiple nucleopolyhedrovirus. Biol. Control.

[53] Williams T., Melo-Molina G.D.C., Jiménez-Fernández J.A., Weissenberger H., Gómez-Díaz J.S., Navarro-de-la-Fuente L., Richards A.R. (2023). Presence of Spodoptera frugiperda multiple nucleopolyhedrovirus (SfMNPV) occlusion bodies in maize field soils of Mesoamerica. Insects.

[54] Clavijo G., Williams T., Muñoz D., Caballero P., López-Ferber M. (2010). Mixed genotype transmission bodies and virions contribute to the maintenance of diversity in an insect virus. Proc. R. Soc. B Biol. Sci..

[55] Graham R.I., Tyne W.I., Possee R.D., Sait S.M., Hails R.S. (2004). Genetically variable nucleopolyhedroviruses isolated from spatially separate populations of the winter moth *Operophtera brumata* (Lepidoptera: Geometridae) in Orkney. J. Invertebr. Pathol..

[56] Hinsberger A., Blachère-López C., López-Ferber M. (2020). Promoting mixed genotype infections in CpGV: Analysis on field and laboratory sprayed apple leaves. Biocontrol Sci. Technol..

[57] Rezapanah M., Shojai-Estabragh S., Huber J., Jehle J.A. (2008). Molecular and biological characterization of new isolates of Cydia pomonella granulovirus from Iran. J. Pest Sci..

[58] Ferrelli M.L., Salvador R. (2023). Effects of mixed baculovirus infections in biological control: A comprehensive historical and technical analysis. Viruses.

[59] Cheng X.W., Carner G.R., Lange M., Jehle J.A., Arif B.M. (2005). Biological and molecular characterization of a multicapsid nucleopolyhedrovirus from *Thysanoplusia orichalcea* (L.) (Lepidoptera: Noctuidae). J. Invertebr. Pathol..

[60] Rincon Castro M.C.D., Ibarra J.E. (1995). Caracterizacion de cepas silvestres de virus de poliedrosis nuclear aisladas de *Trichoplusia ni* (Lepidoptera: Noctuidae) en el centro de México. Vedalia.

[61] Lauzon H.A., Jamieson P.B., Krell P.J., Arif B.M. (2005). Gene organization and sequencing of the Choristoneura fumiferana defective nucleopolyhedrovirus genome. J. Gen. Virol..

[62] Rodríguez V.A., Belaich M.N., Quintana G., Cap A.S., Ghiringhelli P.D. (2012). Isolation and characterization of a nucleopolyhedrovirus from *Rachiplusia nu* (Guenée) (Lepidoptera: Noctuidae). Int. J. Virol. Mol. Biol..

[63] Jakubowicz V., Taibo C.B., Sciocco-Cap A., Arneodo J.D. (2019). Biological and molecular characterization of Rachiplusia nu single nucleopolyhedrovirus, a promising biocontrol agent against the South American soybean pest *Rachiplusia nu*. J. Invertebr. Pathol..

[64] Decker-Franco C., Taibo C.B., Di Rienzo J.A., Alfonso V., Arneodo J.D. (2021). Comparative pathogenesis of generalist AcMNPV and specific RanuNPV in larvae of *Rachiplusia nu* (Lepidoptera: Noctuidae) following single and mixed inoculations. J. Econ. Entomol..

[65] Clavijo G., Williams T., Muñoz D., López-Ferber M., Caballero P. (2009). Entry into midgut epithelial cells is a key step in the selection of genotypes in a nucleopolyhedrovirus. Virol. Sin..

[66] Zwart M.P., van Oers M.M., Cory J.S., van Lent J.W., van der Werf W., Vlak J.M. (2008). Development of a quantitative real-time PCR for determination of genotype frequencies for studies in baculovirus population biology. J. Virol. Meth..

[67] Zwart M.P., Elena S.F. (2015). Matters of size: Genetic bottlenecks in virus infection and their potential impact on evolution. Annu. Rev. Virol..

[68] Washburn J.O., Kirkpatrick B.A., Haas-Stapleton E., Volkman L.E. (1998). M2R enhances Autographa californica M nucleopolyhedrovirus infection of *Trichoplusia ni* and *Heliothis virescens* by preventing sloughing of infected midgut epithelial cells. Biol. Control.

[69] Burden J.P., Possee R.D., Sait S.M., King L.A., Hails R.S. (2006). Phenotypic and genotypic characterisation of persistent baculovirus infections in populations of the cabbage moth (*Mamestra brassicae*) within the British Isles. Arch. Virol..

[70] Vilaplana L., Wilson K., Redman E.M., Cory J.S. (2010). Pathogen persistence in migratory insects: High levels of vertically-transmitted virus infection in field populations of the African armyworm. Evol. Ecol..

[71] Wilson K., Grzywacz D., Cory J.S., Donkersley P., Graham R.I. (2021). Trans-generational viral transmission and immune priming are dose-dependent. J. Anim. Ecol..

[72] Qin F., Xu C., Hu J., Lei C., Zheng Z., Peng K., Wang H., Sun X. (2019). Dissecting the cell entry pathway of baculovirus by single-particle tracking and quantitative electron microscopic analysis. J. Virol..

[73] Beperet I., Irons S., Simón O., King L.A., Williams T., Possee R.D., López-Ferber M., Caballero P. (2014). Superinfection exclusion in alphabaculovirus infections is concomitant with actin reorganization. J. Virol..

[74] Bull J.C., Godfray H.C.J., O’Reilly D.R. (2001). Persistence of an occlusion-negative recombinant nucleopolyhedrovirus in *Trichoplusia ni* indicates high multiplicity of cellular infection. Appl. Environ. Microbiol..

[75] Fu Q.-M., Fang Z., Ren L., Wu Q.-S., Zhang J.-B., Liu Q.-P., Tan L.-T., Weng Q.-B. (2024). Partial Alleviation of Homologous Superinfection Exclusion of SeMNPV Latently Infected Cells by G1 Phase Infection and G2/M Phase Arrest. Viruses.

[76] Sanjuán R. (2017). Collective infectious units in viruses. Trends Microbiol..

[77] Adams J.R., McClintock J.T., Adams J.R., Bonami J.R. (1991). Baculoviridae. part II. Nuclear polyhedrosis viruses of insects. Atlas of Invertebrate Viruses.

[78] Sanjuán R. (2021). The social life of viruses. Annu. Rev. Virol..

[79] Falcon L.A., Hess R.T. (1985). Electron microscope observations of multiple occluded virions in the granulosis virus of the codling moth, *Cydia pomonella*. J. Invertebr. Pathol..

[80] Sciocco de Cap A., Parola A.D., Goldberg A.V., Ghiringhelli P.D., Romanowski V. (2001). Characterization of a granulovirus isolated from *Epinotia aporema* Wals. (Lepidoptera: Tortricidae) larvae. Appl. Environ. Microbiol..

[81] Hamblin M., van Beek N.A.M., Hughes P.R., Wood H.A. (1990). Co-occlusion and persistence of a baculovirus mutant lacking the polyhedrin gene. Appl. Environ. Microbiol..

[82] van Beek N.A.M., Wood H.A., Hughes P.R. (1988). Quantitative aspects of nuclear polyhedrosis virus infections in lepidopterous larvae: The dose-survival time relationship. J. Invertebr. Pathol..

[83] Arrizubieta M., Simón O., Ricarte-Bermejo A., López-Ferber M., Williams T., Caballero P. (2022). Coocclusion of Helicoverpa armigera single nucleopolyhedrovirus (HearSNPV) and Helicoverpa armigera multiple nucleopolyhedrovirus (HearMNPV): Pathogenicity and stability in homologous and heterologous hosts. Viruses.

[84] Munsamy T., Bouwer G. (2020). Determination of the virulence of single nucleopolyhedrovirus occlusion bodies using a novel laser capture microdissection method. J. Gen. Virol..

[85] Hinsberger A., Graillot B., Blachère-López C., Juliant S., Cerutti M., King L.A., Possee R.D., Gallardo F., López-Ferber M. (2020). Tracing baculovirus AcMNPV infection using a real-time method based on ANCHOR™ DNA labeling technology. Viruses.

[86] Fujimoto S., Kokusho R., Kakemizu H., Izaku T., Katsuma S., Iwashita Y., Kawasaki H., Iwanaga M. (2017). Characterization of a Bombyx mori nucleopolyhedrovirus variant isolated in Laos. J. Insect Biotechnol. Sericol..

[87] Beperet I., Barrera G., Simón O., Williams T., López-Ferber M., Caballero P. (2013). The *sf32* unique gene of Spodoptera frugiperda multiple nucleopolyhedrovirus (SfMNPV) is a non-essential gene that could be involved in nucleocapsid organization in occlusion-derived virions. PLoS ONE.

[88] Cheng R.-L., Xu Y.-P., Zhang C.-X. (2012). Genome sequence of a Bombyx mori nucleopolyhedrovirus strain with cubic occlusion bodies. J. Virol..

[89] Li S.N., Wang J.Y., Yuan M.J., Yang K. (2014). Disruption of the baculovirus core gene *ac78* results in decreased production of multiple nucleocapsid-enveloped occlusion-derived virions and the failure of primary infection in vivo. Virus Res..

[90] Yu I.L., Bray D., Lin Y.C., Lung O. (2009). Autographa californica multiple nucleopolyhedrovirus ORF 23 null mutant produces occlusion-derived virions with fewer nucleocapsids. J. Gen. Virol..

[91] Rohrmann G.F. (2014). Baculovirus nucleocapsid aggregation (MNPV vs SNPV): An evolutionary strategy, or a product of replication conditions?. Virus Genes.

[92] Freedman A.S., Huang A.Y., Dixon K.P., Polivka C., Dwyer G. (2024). Effects of host-tree foliage on polymorphism in an insect pathogen. bioRxiv.

[93] Granados R.R., Lawler K.A. (1981). In vivo pathway of Autographa californica baculovirus invasion and infection. Virology.

[94] Washburn J.O., Lyons E.H., Haas-Stapleton E.J., Volkman L.E. (1999). Multiple nucleocapsid packaging of Autographa californica nucleopolyhedrovirus accelerates the onset of systemic infection in *Trichoplusia ni*. J. Virol..

[95] Ikeda M., Yamada H., Hamajima R., Kobayashi M. (2013). Baculovirus genes modulating intracellular innate antiviral immunity of lepidopteran insect cells. Virology.

[96] Nagamine T. (2022). Apoptotic arms races in insect-baculovirus coevolution. Physiol. Entomol..

[97] Beperet I., Simón O., López-Ferber M., Van Lent J., Williams T., Caballero P. (2021). Mixtures of insect pathogenic viruses in a single virion: Towards the development of custom designed insecticides. Appl. Environ.Microbiol..

[98] Pazmiño-Ibarra V., Herrero S., Sanjuán R. (2022). Spatially segregated transmission of co-occluded baculoviruses limits virus–virus interactions mediated by cellular coinfection during primary infection. Viruses.

[99] Xia J., Fei S., Huang Y., Lai W., Yu Y., Liang L., Wu H., Swevers L., Sun J., Feng M. (2024). Single-nucleus sequencing of silkworm larval midgut reveals the immune escape strategy of BmNPV in the midgut during the late stage of infection. Insect Biochem. Mol. Biol..

[100] Nagamine T., Kawasaki Y., Abe A., Matsumoto S. (2008). Nuclear marginalization of host cell chromatin associated with expansion of two discrete virus-induced subnuclear compartments during baculovirus infection. J. Virol..

[101] Cory J.S., Clarke E.E., Brown M.L., Hails R.S., O’Reilly D.R. (2004). Microparasite manipulation of an insect: The influence of the *egt* gene on the interaction between a baculovirus and its lepidopteran host. Funct. Ecol..

[102] Fleming-Davies A.E., Dukic V., Andreasen V., Dwyer G. (2015). Effects of host heterogeneity on pathogen diversity and evolution. Ecol. Lett..

[103] Fleming-Davies A.E., Dwyer G. (2015). Phenotypic variation in overwinter environmental transmission of a baculovirus and the cost of virulence. Am. Nat..

[104] Páez D.J., Fleming-Davies A.E. (2020). Understanding the evolutionary ecology of host–pathogen interactions provides insights into the outcomes of insect pest biocontrol. Viruses.

[105] Lepore L.S., Roelvink P.R., Granados R.R. (1996). Enhancin, the granulosis virus protein that facilitates nucleopolyhedrovirus (NPV) infections, is a metalloprotease. J. Invertebr. Pathol..

[106] Hodgson D.J., Hitchman R.B., Vanbergen A.J., Hails R.S., Possee R.D., Cory J.S. (2004). Host ecology determines the relative fitness of virus genotypes in mixed-genotype nucleopolyhedrovirus infections. J. Evol. Biol..

[107] Boogaard B., Van Oers M.M., Van Lent J.W.M. (2018). An advanced view on baculovirus *per os* infectivity factors. Insects.

[108] Arrizubieta M., Simón O., Williams T., Caballero P. (2015). A novel binary mixture of Helicoverpa armigera single nucleopolyhedrovirus genotypic variants has improved insecticidal characteristics for control of cotton bollworms. Appl. Environ. Microbiol..

[109] del Angel C., Lasa R., Rodríguez del Bosque L.A., Mercado G., Beperet I., Caballero P., Williams T. (2018). Anticarsia gemmatalis nucleopolyhedrovirus from soybean crops in Tamaulipas, Mexico: Diversity and insecticidal characteristics of individual variants and their co-occluded mixtures. Fla. Entomol..

[110] López-Ferber M., Simón O., Williams T., Caballero P. (2003). Defective or effective? Mutualistic interactions between virus genotypes. Proc. R. Soc. Lond. B. Biol. Sci..

[111] Clavijo G., Williams T., Simón O., Muñoz D., Cerutti M., López-Ferber M., Caballero P. (2009). Mixtures of complete and *pif1*- and *pif2*-deficient genotypes are required for increased potency of an insect nucleopolyhedrovirus. J. Virol..

[112] Simón O., Williams T., Cerutti M., Caballero P., López-Ferber M. (2013). Expression of a peroral infection factor determines pathogenicity and population structure in an insect virus. PLoS ONE.

[113] Hinsberger A., Blachère-López C., Knox C., Moore S., Marsberg T., López-Ferber M. (2021). CpGV-M replication in type I resistant insects: Helper virus and order of ingestion are important. Viruses.

[114] Erez Z., Steinberger-Levy I., Shamir M., Doron S., Stokar-Avihail A., Peleg Y., Melamed S., Leavitt A., Savidor A., Albeck S. (2017). Communication between viruses guides lysis-lysogeny decisions. Nature.

[115] Graillot B., Bayle S., Blachère-López C., Besse S., Siegwart M., López-Ferber M. (2016). Biological characteristics of experimental genotype mixtures of Cydia pomonella granulovirus (CpGV): Ability to control susceptible and resistant pest populations. Viruses.

[116] Gueli-Alletti G., Sauer A.J., Weihrauch B., Fritsch E., Undorf-Spahn K., Wennmann J.T., Jehle J.A. (2017). Using next generation sequencing to identify and quantify the genetic composition of resistance-breaking commercial isolates of Cydia pomonella granulovirus. Viruses.

[117] Belda I.M., Beperet I., Williams T., Caballero P. (2019). Genetic variation and biological activity of two closely related alphabaculoviruses during serial passage in permissive and semi-permissive heterologous hosts. Viruses.

[118] Graillot B., Blachère-López C., Besse S., Siegwart M., López-Ferber M. (2016). Host range extension of Cydia pomonella granulovirus: Adaptation to oriental fruit moth, *Grapholita molesta*. Biocontrol.

[119] Hitchman R.B., Hodgson D.J., King L.A., Hails R.S., Cory J.S., Possee R.D. (2007). Host mediated selection of pathogen genotypes as a mechanism for the maintenance of baculovirus diversity in the field. J. Invertebr. Pathol..

[120] Kolodny-Hirsch D.M., Van Beek N.A.M. (1997). Selection of a morphological variant of Autographa californica nuclear polyhedrosis virus with increased virulence following serial passage in *Plutella xylostella*. J. Invertebr. Pathol..

[121] Molina-Ruiz C.S., Zamora-Briseño J.A., Simón O., Lasa R., Williams T. (2024). A qPCR assay for the quantification of selected genotypic variants of Spodoptera frugiperda multiple nucleopolyhedrovirus (Baculoviridae). Viruses.

[122] Kennedy D.A., Dwyer G. (2018). Effects of multiple sources of genetic drift on pathogen variation within hosts. PLoS Biol..

[123] van der Werf W., Hemerik L., Vlak J.M., Zwart M.P. (2011). Heterogeneous host susceptibility enhances prevalence of mixed-genotype micro-parasite infections. PLoS Comput. Biol..

[124] Hudson A.I., Fleming-Davies A.E., Páez D.J., Dwyer G. (2016). Genotype-by-genotype interactions between an insect and its pathogen. J. Evol. Biol..

[125] Siegwart M., Maugin S., Besse S., López-Ferber M., Hinsberger A., Gauffre B. (2020). Le carpocapse des pommes résiste au virus dela granulose. Phytoma.

[126] Murillo R., Muñoz D., Ruíz-Portero M.C., Alcázar M.D., Belda J.E., Williams T., Caballero P. (2007). Abundance and genetic structure of nucleopolyhedrovirus populations in greenhouse substrate reservoirs. Biol. Control.

[127] Martínez-Solís M., Collado M.C., Herrero S. (2020). Influence of diet, sex, and viral infections on the gut microbiota composition of *Spodoptera exigua* caterpillars. Front. Microbiol..

[128] Shikano I. (2017). Evolutionary ecology of multitrophic interactions between plants, insect herbivores and entomopathogens. J. Chem. Ecol..

[129] Hodgson D.J., Vanbergen A.J., Hartley S.E., Hails R.S., Cory J.S. (2002). Differential selection of baculovirus genotypes mediated by different species of host food plant. Ecol. Lett..

[130] Raymond B., Vanbergen A., Pearce I., Hartley S., Cory J., Hails R. (2002). Host plant species can influence the fitness of herbivore pathogens: The winter moth and its nucleopolyhedrovirus. Oecologia.

[131] Cory J.S., Myers J.H. (2009). Within and between population variation in disease resistance in cyclic populations of western tent caterpillars: A test of the disease defence hypothesis. J. Anim. Ecol..

[132] Lee H.H., Miller L.K. (1978). Isolation of genotypic variants of Autographa californica nuclear polyhedrosis virus. J. Virol..

[133] Lynn D.E., Shapiro M., Dougherty E.M. (1993). Selection and screening of clonal isolates of the Abington strain of gypsy moth nuclear polyhedrosis virus. J. Invertebr. Pathol..

[134] Anonymous (2019). AgBiTech launches lepidopteran biocontrol options. Outlooks Pest Manag..

[135] Popham H.J.R., Nusawardani T., Bonning B.C., Murhammer D. (2016). Introduction to the use of baculoviruses as biological insecticides. Baculovirus and Insect Cell Expression Protocols.

[136] Kroemer J.A., Bonning B.C., Harrison R.L. (2015). Expression, delivery and function of insecticidal proteins expressed by recombinant baculoviruses. Viruses.

[137] Williams T., López-Ferber M., Caballero P. (2022). Nucleopolyhedrovirus coocclusion technology: A new concept in the development of biological insecticides. Front. Microbiol..

[138] Caballero P., Williams T., Muñoz-Labiano D., Murillo-Perez R., Lasa-Covarrubias R. (2006). Nuevos Genotipos del Nucleopoliedrovirus de Spodoptera exigua y Uso de los Mismos en el Control de las Plagas Producidas por Este Insecto.

[139] Carballo A., Murillo R., Jakubowska A., Herrero S., Williams T., Caballero P. (2017). Co-infection with iflaviruses influences the insecticidal properties of Spodoptera exigua multiple nucleopolyhedrovirus occlusion bodies: Implications for the production and biosecurity of baculovirus insecticides. PLoS ONE.

[140] Briese D.T. (1982). Genetic basis for resistance to a granulosis virus in the potato tuber moth, *Phthorimaea operculella*. J. Invertebr. Pathol..

[141] Abot A.R., Moscardi F., Fuxa J.R., Sosa-Gomez D.R., Richter A.R. (1996). Development of resistance by *Anticarsia gemmatalis* from Brazil and the United States to a nuclear polyhedrosis virus under laboratory selection pressure. Biol. Control.

[142] Fuxa J.R., Richter A.R. (1989). Reversion of resistance by *Spodoptera frugiperda* to nuclear polyhedrosis virus. J. Invertebr. Pathol..

[143] Martínez A.M., Caballero P., Villanueva M., Miralles N., San Martín I., López E., Williams T. (2004). Formulation with an optical brightener does not increase probability of developing resistance to Spodoptera frugiperda nucleopolyhedrovirus in the laboratory. J. Econ. Entomol..

[144] Nakai M., Takahashi K., Iwata K., Tanaka K., Koyanagi J., Ookuma A., Takatsuka J., Okuno S., Kunimi Y. (2017). Acquired resistance to a nucleopolyhedrovirus in the smaller tea tortrix *Adoxophyes honmai* (Lepidoptera: Tortricidae) after selection by serial viral administration. J. Invertebr. Pathol..

[145] Moscardi F. (1999). Assessment of the application of baculoviruses for control of Lepidoptera. Annu. Rev. Entomol..

[146] Tanada Y. (1964). A granulosis virus of the codling moth, *Carpocapsa pomonella* (Linnaeus) (Olethreutidae, Lepidoptera). J. Insect Pathol..

[147] Fritsch E., Undorf-Spahn K., Kienzle J., Zebitz C., Huber J. (2005). Apfelwickler-Granulovirus: Erste Hinweise auf Unterschiede in der Empfindlichkeit lokaler Apfelwicklerpopulationen. Nachr Dt Pflanzenschutzdienstes.

[148] Sauphanor B., Berling M., Toubon J.F., Reyes M., Delnatte J., Allemoz P. (2006). Cases of resistance to granulosis virus in the codling moth. Phytoma.

[149] Eberle K.E., Sayed S., Rezapanah M., Shojai-Estabragh S., Jehle J.A. (2009). Diversity and evolution of the Cydia pomonella granulovirus. J. Gen. Virol..

[150] Sauer A.J., Fritsch E., Undorf-Spahn K., Nguyen P., Marec F., Heckel D.G., Jehle J.A. (2017). Novel resistance to Cydia pomonella granulovirus (CpGV) in codling moth shows autosomal and dominant inheritance and confers cross-resistance to different CpGV genome groups. PLoS ONE.

[151] Feng G., Thumbi D.K., de Jong J., Hodgson J.J., Arif B., Doucet D., Krell P.J. (2012). Selection and characterization of Autographa californica multiple nucleopolyhedrovirus DNA polymerase mutations. J. Virol..

[152] Baillie V.L., Bouwer G. (2013). The effect of inoculum dose on the genetic diversity detected within Helicoverpa armigera nucleopolyhedrovirus populations. J. Gen. Virol..

[153] Díaz-Muñoz S.L., Sanjuán R., West S. (2017). Sociovirology: Conflict, cooperation, and communication among viruses. Cell Host Microbe.

[154] Leeks A., Bono L.M., Ampolini E.A., Souza L.S., Höfler T., Mattson C.L., Dye A.E., Díaz-Muñoz S.L. (2023). Open questions in the social lives of viruses. J. Evol. Biol..

[155] Cory J.S. (2017). Evolution of host resistance to insect pathogens. Curr. Opin. Insect Sci..

[156] Zheng-Li L.Y. (2017). The Role of Pathogen Diversity on the Evolution of Resistance. Master’s Thesis.

[157] Visher E., Uricchio L., Bartlett L., de Namur N., Yarcan A., Alhassani D., Boots M. (2022). The evolution of host specialization in an insect pathogen. Evolution.

[158] Bartlett L.J., Wilfert L., Boots M. (2022). XA genotypic trade-off between constitutive resistance to viral infection and host growth rate. Evolution.

[159] Boots M. (2011). The evolution of resistance to a parasite is determined by resources. Am. Nat..

[160] Roberts K.E., Meaden S., Sharpe S., Kay S., Doyle T., Wilson D., Bartlett L.J., Paterson S., Boots M. (2020). Resource quality determines the evolution of resistance and its genetic basis. Mol. Ecol..

[161] Karlsson-Green K. (2021). The effects of host plant species and larval density on immune function in the polyphagous moth *Spodoptera littoralis*. Ecol. Evol..

[162] Undorf-Spahn K., Fritsch E., Huber J., Kienzle J., Zebitz C.P.W., Jehle J.A. (2012). High stability and no fitness costs of the resistance of codling moth to Cydia pomonella granulovirus (CpGV-M). J. Invertebr. Pathol..

