# Correction: López-Ferber et al. Baculovirus Genetic Diversity and Population Structure. *Viruses* 2025, *17*, 142

**DOI:** 10.3390/v17050683

**Published:** 2025-05-07

**Authors:** Miguel López-Ferber, Primitivo Caballero, Trevor Williams

**Affiliations:** 1 HSM, University Montpellier, IMT Mines Ales, CNRS, IRD, 30319 Alès, France; 2 Institute for Multidisciplinary Research in Applied Biology, Universidad Pública de Navarra, 31006 Pamplona, Spain; pcm92@unavarra.es; 3 Departamento de Investigación y Desarrollo, Bioinsectis SL, 31110 Noain, Spain; 4 Instituto de Ecología AC (INECOL), Xalapa, Veracruz 91073, Mexico

## Missing Funding Information

In the original publication [1], the funder Ministerio de Ciencia e Innovación—AGL2017-83498-C2-1-R, PID2020-117062RB-C21, and PID2023-152494OB-I00—of Primitivo Caballero was not indicated. The authors state that the scientific conclusions are unaffected. This correction was approved by the Academic Editor. The original publication has also been updated.
